# Clinical Applications of Classical and Novel Biological Markers of Pancreatic Cancer

**DOI:** 10.3390/cancers14081866

**Published:** 2022-04-07

**Authors:** Leonel Pekarek, Oscar Fraile-Martinez, Cielo Garcia-Montero, Miguel A. Saez, Ines Barquero-Pozanco, Laura del Hierro-Marlasca, Patricia de Castro Martinez, Adoración Romero-Bazán, Miguel A. Alvarez-Mon, Jorge Monserrat, Natalio García-Honduvilla, Julia Buján, Melchor Alvarez-Mon, Luis G. Guijarro, Miguel A. Ortega

**Affiliations:** 1 Department of Medicine and Medical Specialities, Faculty of Medicine and Health Sciences, University of Alcalá, 28801 Alcala de Henares, Spain; leonel.pakarek@edu.uah.es (L.P.); cielo.garcia@edu.uah.es (C.G.-M.); msaega1@oc.mde.es (M.A.S.); ines.barquero@edu.uah.es (I.B.-P.); l.hierro@edu.uah.es (L.d.H.-M.); patricia.castrom@edu.uah.es (P.d.C.M.); arb00031@red.ujaen.es (A.R.-B.); miguangel.alvarez@edu.uah.es (M.A.A.-M.); jorge.monserrat@uah.es (J.M.); natalio.garcia@uah.es (N.G.-H.); mjulia.bujan@uah.es (J.B.); mademons@uah.es (M.A.-M.); 2 Ramón y Cajal Institute of Sanitary Research (IRYCIS), 28034 Madrid, Spain; luis.gonzalez@uah.es; 3 Oncology Service, Guadalajara University Hospital, 19002 Guadalajara, Spain; 4 Pathological Anatomy Service, Central University Hospital of Defence-UAH Madrid, 28801 Alcala de Henares, Spain; 5 Immune System Diseases-Rheumatology, Oncology Service an Internal Medicine (CIBEREHD), University Hospital Príncipe de Asturias, 28806 Alcala de Henares, Spain; 6 Unit of Biochemistry and Molecular Biology, Department of System Biology (CIBEREHD), University of Alcalá, 28801 Alcala de Henares, Spain; 7 Cancer Registry and Pathology Department, Principe de Asturias University Hospital, 28806 Alcala de Henares, Spain

**Keywords:** histological markers, pancreatic adenocarcinoma, pancreatic immunohistochemistry, serological markers

## Abstract

**Simple Summary:**

Pancreatic cancer is one of the most heterogeneous and aggressive tumours that exist. The median survival of diagnosed patients is limited. For all these reasons, it is necessary to develop new disease markers that allow guidelines for clinical action to improve the average survival of patients with pancreatic cancer.

**Abstract:**

The incidence and prevalence of pancreatic adenocarcinoma have increased in recent years. Pancreatic cancer is the seventh leading cause of cancer death, but it is projected to become the second leading cause of cancer-related mortality by 2040. Most patients are diagnosed in an advanced stage of the disease, with very limited 5-year survival. The discovery of different tissue markers has elucidated the underlying pathophysiology of pancreatic adenocarcinoma and allowed stratification of patient risk at different stages and assessment of tumour recurrence. Due to the invasive capacity of this tumour and the absence of screening markers, new immunohistochemical and serological markers may be used as prognostic markers for recurrence and in the study of possible new therapeutic targets because the survival of these patients is low in most cases. The present article reviews the currently used main histopathological and serological markers and discusses the main characteristics of markers under development.

## 1. Introduction

The epidemiology and prognosis of pancreatic adenocarcinoma are fundamental to understanding its increasing importance in oncology, and it has become one of the main challenges in the development of new therapies and diagnosis of digestive system tumours. Histologically, pancreatic adenocarcinoma represents approximately 90% of pancreatic cancers and is one of the main causes of oncological mortality, causing more than 400,000 deaths per year worldwide despite being the seventh most frequent cancer. [1] The increase in incidence, mortality, and difficulty of developing effective therapies have led to the prediction that pancreatic cancer will become the second leading cause of death from cancer in developed countries by 2040. [2] Pancreatic cancer in developed countries has a global incidence of approximately six patients per 100,000 inhabitants. [3] Although there are many approaches for the treatment of pancreatic adenocarcinoma (e.g., surgery, chemotherapy, and radiotherapy), mortality has not decreased as much as other types of malignant neoplasms. [4] Most patients are in an advanced stage at the time of diagnosis. The survival rate 5 years after diagnosis is less than 10% in different studies, with a mean survival of 6 months because it is a disseminated disease with metastasis in multiple organs. [5,6] Several risk factors increase the probability and obscure the prognosis in these patients, including obesity, tobacco, alcohol, and premalignant lesions, which act in a multifactorial manner. However, there are no effective screening programs that reduce mortality or improve survival in high-risk patients [7].

The current therapeutic options for the management of pancreatic adenocarcinoma include tumour resection using different surgical techniques or the application of different chemotherapeutic regimens with gemcitabine and gemcitabine derivatives, such as Abraxane, in combination with other chemotherapeutic agents, such as fluorouracil and oxaliplatin. However, few patients are diagnosed in early stages of the disease, and chemotherapy exhibits moderate efficacy. [8] The utility of radiotherapy and immunotherapy is being evaluated in different clinical trials. Notably, most patients have disseminated metastatic disease at the time of diagnosis. Numerous metabolic pathways were described in recent years, and a wide variety of immunohistochemical and serological markers for daily clinical practice are in development to establish prognostic factors for screening and evaluating the response to therapeutic targets. The study of prognostic factors is especially useful when approaching patients in advanced stages because the use of prognostic factors would allow for stratification of possible treatments and planning for palliative care to improve the doctor–patient relationship and stage patients correctly to prevent metastatic recurrence.

The present article describes the novelties, updates, and future perspectives of serological and immunohistochemical markers for the diagnosis, treatment, and management of patients with pancreatic adenocarcinoma.

## 2. CA19-9

The serological marker CA19-9, or sialized Lewis antigen, is the most used marker in daily clinical practice in the diagnosis and monitoring of pancreatic adenocarcinoma. Despite having variable sensitivity and specificity (up to 92% according to various authors), the usefulness of CA19-9 lies in its diagnostic value in disseminated disease. The best cut-off to differentiate pancreatic adenocarcinoma from other benign and malignant entities is 37 U/mL. Its positive predictive value is only 0.9% in the asymptomatic population [9,10,11]. Another limitation of this protein is that 5–10% of the Caucasian population does not express it, and thus they do not present elevated CA19-9 in blood [12]. This antigen level in the blood is also related to tumour size, and its positive predictive value is substantially lower for small neoplasms and asymptomatic patients [13]. CA19-9 is elevated in a multitude of neoplastic and non-neoplastic pathologies, such as cholangiocarcinoma, hepatobiliary infections, pulmonary fibrosis, and endometriosis, which lead to falsely high values that are not related to true pancreatic neoplasia [14].

The degree of CA19-9 elevation is related to the prognosis, with a direct correlation between its levels and tumour burden, but it will not rise until advanced stages when the tumour has grown, and there is a large metastatic burden [15]. Numerous authors described cut-off points of CA19-9 values for predicting hidden metastatic disease. For example, Maithe et al. indicated that values above 130 U/mL of CA19-9 in patients who are candidates for total resection had a high probability of broad metastatic occult dissemination that limits the benefit of surgical resection with curative intent [16]. Frequent monitoring, every 1–3 months, in patients on active chemotherapeutic treatment with disseminated disease allowed for the detection of locoregional or metastatic relapses and the selection of patients who required additional imaging tests. George et al. demonstrated its clinical utility in 6118 patients with pancreatic adenocarcinoma, which translated into better management and monitoring of these patients [17]. Therefore, CA19-9 is useful in advanced stages and relapse but not in early diagnosis or population screening due to its limited detectability in early stages of the disease. This deficiency supports the need to analyse histological and serological markers that improve the diagnostic yield of CA19-9 and may be used to better stratify patients at risk who can participate in screening programmes.

## 3. Other Promising Serological Markers Used in Pancreatic Cancer

The difficulty of establishing a correct diagnosis in pancreatic adenocarcinoma is based on the study of fine-needle biopsy guided by ultrasound and the elevation of the CA19-9 marker in peripheral blood, which is not elevated until advanced stages and may be elevated by other malignant or inflammatory neoplasms [18]. New serological markers that improve early diagnosis in these patients were discovered in recent years, and their clinical utility has been studied using receiver operating characteristic (ROC) curves comparing patients with pancreatic adenocarcinoma, patients with preinvasive pancreatic lesions, and healthy controls. For example, Takayama et al. studied the use of the serological tumour antigen regenerating islet-derived protein 4 (REG4) in 200 subjects, including patients with adenocarcinoma, intrapapillary mucinous tumours, patients with pancreatitis, and healthy controls. The combination of CA19-9 and REG4 had a diagnostic sensitivity of 100% and a specificity of 60%. The area under the ROC curve (AUC) of REG4 reached a value of 0.922, compared to 0.884 for CA19-9, in patients with differential pancreatic adenocarcinoma, even at early stages, compared to healthy controls [19]. The AUC, with a maximum value of 1, demonstrates the diagnostic capacity in the sensitivity and specificity of a diagnostic test. Zinczuk et al. demonstrated that the increases in carcinoembryonic-antigen-related cell adhesion molecule (CEACAM) 1, CEACAM5, and CEACAM6 in histological samples of patients with pancreatic adenocarcinoma were associated with a worse prognosis [20]. Simeone et al. found elevated CEACAM1 in serological samples of 195 patients (81 patients with pancreatic adenocarcinoma, 53 with chronic pancreatitis, and 61 healthy controls) compared to the other patients. The combination of CEACAM1 and CA19-9 had an AUC of 0.948 for distinguishing healthy controls from patients with adenocarcinoma. CEACAM1 also distinguished between chronic pancreatitis and pancreatic adenocarcinoma patients with an AUC of 0.752. [21]

Identifying the initial stages and knowing how to differentiate pancreatic cancer from other entities, such as chronic pancreatitis, are fundamental in the diagnosis of pancreatic cancer. Poruk et al. found the clinical utility of serological levels of osteopontin and tissue inhibitor metalloproteinase 1 (TIMP1) in differentiating patients with chronic pancreatitis, pancreatic adenocarcinoma of different stages, and no pancreatic disease in 200 patients. These authors studied the possibility of implementing a diagnostic algorithm using CA19-9, OPN, and TIMP1 and found a diagnostic accuracy of 89.5% in differentiating patients with pancreatic adenocarcinoma, patients with chronic pancreatitis, and healthy people. In addition to the diagnostic utility, there was an inverse relationship between OPN and TIMP1 levels in serum and survival, which may also be used as a prognostic factor [22]. The diagnostic algorithm allowed better differentiation of patients in early stages than advanced stages, which may be used to better stratify patients who are candidates for curative surgery or neoadjuvant chemotherapy. The serological elevation of TIMP1 was related to a worse prognosis in patients with other digestive neoplasms, such as colon cancer or gastric cancer [23,24]. TIMP1 is involved in the activation of CD63, which leads to histological alterations in hepatic stellate cells that cause cellular remodelling and promotion of the presence of metastatic niches [25]. Prokopchuk et al. correlated TIMP1 to clinical markers of cachexia and jaundice, such as ferritin haemoglobin, weight loss, and spirometric alterations, which are sources of many comorbidities in patients with pancreatic adenocarcinoma. Their research shows that in patients with jaundice, TIMP-1 is not useful as a prognostic or diagnostic marker because its values could be overestimated in patients with pancreatic adenocarcinoma. However, in patients with cachexia without jaundice, TIMP-1 is useful as a diagnostic marker in pancreatic adenocarcinoma [26].

A derivative of transforming growth factor β (TGF-β), macrophage inhibitory cytokine (MIC1), has been studied as a possible marker of pancreatic adenocarcinoma. Wang et al. examined the relationship between serological and histological levels of MIC1 in 64 patients, including 1571 subjects with pancreatic adenocarcinoma, chronic pancreatitis, benign pancreatic neoplasms, and other malignant neoplasms and healthy controls. This marker may be used in the diagnosis of adenocarcinoma in patients who are negative for CA19-9/Lewis antigen, which is up to 15% of patients. MIC1 had an AUC of 0.886 in patients with CA19-9, which demonstrates its diagnostic utility in pancreatic adenocarcinoma. Its AUC was 0.935 when comparing patients with pancreatic adenocarcinoma versus patients without pancreatic adenocarcinoma but with other benign digestive tumours with higher serological levels in patients with small invasive pancreatic neoplasms [27]. When comparing pancreatic non-invasive neoplasms vs. adenocarcinoma of the pancreas, the combination of MIC1 and CA19-9 allowed better discrimination of the initial stages of pancreatic adenocarcinoma. Therefore, MIC1 may be used to evaluate the success of a curative surgical intervention because its decrease is accompanied by lower locoregional and metastatic recurrence [28].

Hosokawa et al. compared another serological marker, sPan-1, which is a pancreatic adenocarcinoma antigen, to other serological markers, such as CA19-9, carcinoembryonic antigen (CEA), and duke pancreatic monoclonal antigen type 2 (DUPAN-2), in the recurrence of early stages of pancreatic adenocarcinoma. This study followed 172 patients who were candidates for curative resection for 15 years, and the recurrence of pancreatic adenocarcinoma was evaluated. Preoperative elevations of s-pancreas antigen-1 (SPan-1) over 41 U/mL were associated with an early recurrence of locoregional and metastatic disease. Therefore, the therapeutic benefit of curative resection in these patients was limited [29].

Another serological marker that is elevated in up to 60% of patients is CEA [30]. Meng et al. performed a meta-analysis of the diagnostic utility and prognosis of CEA compared to CA19-9, and the AUC of CEA was 90%, which supports its use as a prognostic factor because its elevation was associated with worse prognosis and higher referral rates [31].

Therefore, the usefulness of serological markers other than CA19-9 for the recurrence and early diagnosis of pancreatic adenocarcinoma has been evaluated in a wide variety of studies. A variety of serological markers in adenocarcinoma showed usefulness when combined with CA19-9, which is the most used clinical marker despite a lack of increase in 15% of patients. CA19-9 is not useful in early diagnoses, and it is elevated in many different digestive system neoplasms and benign diseases. Therefore, the implementation of serological diagnostic panels with multiple markers would allow for better stratification of patients with the possibility of recurrence and the differentiation of preinvasive pancreatic neoplasms, which would improve the early diagnosis of pancreatic adenocarcinoma.

## 4. PAM4/MUC5AC

Tumour mucins have been the source of interesting findings in different neoplasms of the digestive tract. Mucins are a set of glycoproteins that are present on the surface of epithelial cells of the digestive tract and produced by goblet cells, which lubricate and acidify the microenvironment to reduce infection by exogenous pathogens via the action of antimicrobial peptides and mediate locoregional immunity. [32]. MUC3 and MUC6 are expressed in nonpathological states in the pancreas [33], and MUC1 and MUC5AC are expressed in pathological situations. MUC5AC has its highest expression in premalignant lesions and pancreatic adenocarcinomas [34]. To understand the clinical utility of this marker, note that before the appearance of adenocarcinoma, there are preinvasive histological grades of dysplasia in pancreatic tissue. Most of these preinvasive neoplasms are detected incidentally and do not lead to true pancreatic adenocarcinoma in patients [35]. It is difficult but important to distinguish between possible pancreatic adenocarcinoma and mucinous tumours because definitive surgical intervention is not exempt from mortality and comorbidities. The locoregional activity of MUC5AC and its importance within pancreatic adenocarcinoma is related to different metabolic pathways that favour metastatic dissemination (e.g., activation of ERK and VEGFR1 or promotion of the activation of metalloproteases and integrins), and it is related to chemoresistance mechanisms to gemcitabine via deregulation of the E-cadherin/β-catenin axis [36,37,38,39]. MUC5AC generates local immunosuppression by inhibiting the ligands associated with TNF-induced apoptosis and decreasing the production of the proinflammatory cytokine CXCL8 [40]. The aetiopathogenesis of MUC5AC production by invasive pancreatic cell lines is not clear, but it was related to alterations of cyclic AMP and altered receptors of the active vasointestinal peptide [41]. Because MUC5AC is present in premalignant lesions (e.g., intraductal mucinous papillary tumours) and invasive lesions, Yang et al. analysed plasma extracellular vesicles from patients with high-grade mucinous papillary tumours. The elevated plasma levels of MUC5AC had higher histological progression and a greater tendency to invade. High serum MUC5AC had 82% sensitivity and 100% specificity in diagnosing high-grade invasive lesions, which demonstrates its utility in the differential diagnosis of mucinous papillary tumours against probable pancreatic adenocarcinomas [42]. Zhang et al. evaluated the combination of CA19-9 with the serological measurement of MUC5AC in 61 patients with pancreatic adenocarcinoma compared to healthy control patients, patients with choledocholithiasis, and patients with chronic pancreatitis. This combination had an AUC of 0.894, which demonstrates its usefulness in the serological diagnosis of this neoplasm [43]. The results of different studies on the prognostic utility of this marker are not conclusive because some authors found better survival rates in patients with the presence of MUC5AC, but later studies showed worse average survival rates [44,45,46]. Therefore, studies of its serological levels and tissue expression using immunohistochemistry should be performed to define whether the prognosis varies depending on the stage.

The therapeutic utility of alterations in mucin was studied using PAM4. PAM4 is an IgG1 monoclonal antibody against MUC1 that binds to the PAM4 epitope in pancreatic adenocarcinoma cells. Gold et al. showed immunoreaction in 48 of 55 patients (87%), which demonstrates its usefulness as a diagnostic marker. Although MUC1 is present in normal pancreatic cells, it has no immunoreactivity with PAM4 [47]. Gold et al. later studied 298 patients with pancreatic cancer of different stages compared to healthy controls, patients with pancreatitis, and patients with other neoplasms and observed 76% sensitivity and 96% specificity for PAM4, which supports its usefulness in the differential diagnosis of benign vs. malignant lesions [48]. The diagnostic utility of PAM4 as a radiomarker in the form of ^131^I-PAM4 or ^111^In-PAM4 was evaluated in different studies, and it showed the detection of microlesions of up to 1 cm, which suggests its use in tumours of small size where the elevation of CA19-9 is not evident. The therapeutic utility of the I-cPAM4 or ^90^Y-DOTA-cPAM4 association with gemcitabine was also studied, and it yielded improvements in survival in mice with human pancreatic adenocarcinoma cell lines [49].

The usefulness of PAM4 and MUC5AC is for screening in early stages and as serological markers of invasion in premalignant lesions. PAM4 may be used in radiological diagnosis, and it is a possible therapeutic target for immunotherapy of pancreatic adenocarcinoma.

## 5. Angiogenesis and Lymphangiogenesis Markers

Angiogenesis and lymphangiogenesis are determinants of tumour growth and the progression and metastatic dissemination of malignant neoplasms. The clinical utility of the study of these processes in pancreatic adenocarcinoma is the possibility of using targeted therapies against them in the same way as breast, colon, or lung tumours, where monoclonal antibodies against vascular endothelial growth factor (VEGF) are used [50].

The pathophysiology of angiogenesis in pancreatic adenocarcinoma is due to genetic and epigenetic alterations with different alterations at the levels of transcription factors, such as Sp1 or NFκB, which cause a series of changes in the angiogenesis process [51,52]. For example, numerous authors described a great variety of proangiogenic factors that involve modifications in fibroblasts that are intimately related to the process of neovascularisation and desmoplasia, such as VEGF, IGF-I, TGF-β, and IL8 [53,54,55]. These changes lead the cells to the pancreatic microenvironment (e.g., stellate cells, tumour cells, fibroblasts, or macrophages), which causes a state of cellular hypoxia and the release of a series of proangiogenic factors. Therefore, the process of angiogenesis is induced at the locoregional level [56]. Histologically, an increase in microscopic vascularization is observed microscopically in different invasive neoplasms of the colon, breast, or lung, which also occurs in pancreatic adenocarcinoma [57]. There is a high microvascular density with a heterogeneous distribution throughout the tumour in pancreatic cancer, which contains histological areas with altered integrity of the blood microvessels [58]. At the macroscopic level, desmoplastic alterations and epithelial–mesenchymal transition cause pancreatic adenocarcinoma to become a hypovascularized tumour with great invasive capacity and a high vascularisation density [59]. This hypovascular environment does not slow tumour growth because the altered cells of the microvessels exhibit modifications in glucose receptors, especially GLUT-1, which allow an optimal nutrient supply to the tumour cells. Hypovascularity also lowers the efficacy of chemotherapeutic regimens by acting as a physical barrier within the chemoresistant tumour [60]. Annese et al. found that these microscopic alterations in tumour vascularisation were related to a higher rate of recurrence and worsening of the prognosis [61].

The most commonly used histological marker in pancreatic adenocarcinoma is CD34, but other markers, such as CD41 and factor VIII, are measured using immunohistochemistry [62]. For example, Takagi et al. analysed vascularisation density in 41 patients using immunohistochemistry for factor VIII and found that an increase in vascularisation density was associated with a worse prognosis and a high rate of local and metastatic recurrence [63].

Therefore, different clinical trials evaluated different therapies directed against the angiogenesis process to limit tumour progression from different points and promote the response to different chemotherapeutic drugs. For example, Berlin et al. evaluated the use of bevacizumab (an anti-VEGF monoclonal antibody) with gemcitabine and radiotherapy in 127 patients with pancreatic adenocarcinoma, and complete tumour resection did not correlate with a better prognosis [64]. Awasthi et al. studied the use of nintedanib, which is an angiokinase inhibitor of VEGFR1–3, fibroblast growth factor receptor 1–3, and platelet-derived growth factor receptor (PDGFR) α/β, in human pancreatic adenocarcinoma cell lines, such as AsPC-1 and BxPC-3, and in vivo in nu/nu mice. Their results in vivo and in vitro showed a decrease in cell proliferation [65]. This strategy is being studied in patients with pancreatic adenocarcinoma in a phase 2 clinical trial (NCT02902484), where the tolerability and safety of the combination of nintedanib with gemcitabine are also being evaluated. Chiorean et al. evaluated the use of sorafenib in 27 patients with advanced pancreatic adenocarcinoma. Their results show a limited therapeutic effect of this combination with little improvement in mean survival [66]. Kindeler et al. observed the same result with the combination of axitinib (selective inhibitor of VEGFR1–3) and gemcitabine in 632 patients, and the combination did not improve the average survival of patients with advanced adenocarcinoma compared to conventional treatment [67].

The role of the lymphatic system in the metastatic progression of pancreatic adenocarcinoma is equally as important as vascular invasion. Different authors showed an increase in tissue levels of VEGF-C and VEGF-D in pancreatic adenocarcinoma [68]. Numerous markers of lymphangiogenesis may be used to demonstrate lymphatic invasion, such as VEGFR3, podoplanin, and D2–40 [69]. D2–40 is a classic marker of lymphangiogenesis because it is expressed in lymphatic tissue but not the vascular endothelium, and it is the preferred marker to evaluate the lymphatic system in pancreatic cancer [70]. The angiogenic process in pancreatic adenocarcinoma is limited to the periphery of the tumour because the presence of lymphatic vessels in the central area of the tumour is very limited due to the destruction of tumour cells and desmoplastic changes in the fibroblast tissue [71,72]. The process of microlymphangiogenesis is related to micrometastases in distal lymph nodes in a wide variety of tumours, including melanoma, gastric adenocarcinoma, and the different varieties of breast malignancies. Understanding this process has allowed authors, such as Wang et al., to relate an increase in the density of lymphatic vessels in the periphery of the tumour with more aggressive neoplasms that exhibited metastatic dissemination and a worse prognosis [73]. This association is why understanding the processes of angiogenesis and lymphangiogenesis may be useful in evaluating the possible use of guided immunotherapy using markers expressed in the tumour itself, which favours metastatic dissemination and limits the action of chemotherapeutic and radiotherapeutic regimens.

## 6. Circulating Tumour Cells

The main characteristic underlying the lethality and fatal outcome of pancreatic adenocarcinoma is its subclinical metastatic capacity, which is difficult to evaluate. Micrometastases in pancreatic adenocarcinoma begin with vascular invasion of the portal circulation and the invasion of peripheral blood. The liver, spleen, and lung are primarily invaded [74]. The measurement of circulating tumour cells was studied in different tumours, such as breast or colorectal cancer, using different materials that demonstrated potential in screening, early diagnosis, and predicting tumour recurrence.

The usefulness of circulating tumour or epithelial cells is that they are not detectable in healthy controls and their possible usefulness in the diagnosis and prognosis of patients with different neoplasms [75]. Over the last few years, the sensitivity of detecting these cells using microfilters has gone from 10% to 90% due to improvements in biomaterials, and the main advantage of their detection is that it is a minimally invasive and repeatable diagnostic mechanism because the main sample is obtained from peripheral blood [76]. Most cases of pancreatic adenocarcinoma require endoscopic or ultrasound-guided biopsy, which may yield negative results in up to 8% of positive cases [77]. As we previously indicated, the presence of new biomaterials, such as microfilters, electrical separation, and the use of antibodies against the epithelial cell adhesion molecule (EpCAM) improves the sensitivity of the detection of these cells because their number in peripheral blood is very limited (approximately one cell per 10 million leukocytes). [78,79] Immunohistochemical markers, microRNA measurement, or mutations in the KRAS gene may be added to improve sensitivity [80]. Their clinical usefulness has been studied primarily as a prognostic factor. For example, Poruk et al. detected a positivity rate of 78% primarily in patients in stages I and II, which demonstrated that patients with circulating cells tended to have lower average survival and worse prognosis [81]. Gemenetzis et al. found a positivity rate of 84% in 200 patients, which demonstrates that a high circulating cell count in patients with any tumour stage was associated with a worse average survival with a higher recurrence rate after neoadjuvant chemotherapy treatment [82]. Geung Son et al. studied the usefulness of the measurement of peripheral blood plectin-1 in stages I and II in pancreatic adenocarcinoma patients and found a positivity rate of approximately 50% of this marker [83].

The usefulness of circulating cells is that they are detectable in blood in the early or preinvasive stages, but their positivity is limited in these stages. Other markers, such as C133, vimentin, and CD44, were also studied, but plectin-1 had similar levels in peripheral serum and blood obtained from portal vessels [84]. Therefore, the usefulness of the measurement of circulating tumour cells depends on the stage. Initial or advanced tests may be used as prognostic factors for recurrence and, to a lesser extent, early diagnosis, but future studies should improve the detection of circulating cells in peripheral blood.

## 7. MicroRNAs and Exosomes

MicroRNAs are small RNA molecules of approximately 20 noncoding nucleotides that regulate many genes posttranscriptionally. Numerous authors described their involvement in different pathways of cellular differentiation, proliferation, and apoptosis and found that they acted as endogenous epigenetic factors promoting or suppressing the expression of a gene after transcription [85]. A microRNA molecule regulates the posttranscription of up to 200 different genes, and their study elucidated the underlying pathophysiology of a wide variety of diseases, and they may be analysed as possible therapeutic targets in a great variety of neoplasms [86].

The effects of a microRNA in a tumour cell depend on the tissue line of the tumour because the same microRNA can act as a stimulator in one tissue and an inhibitor in another [87]. One of the most important characteristics of pancreatic adenocarcinoma is its high chemoresistance, which limits the therapeutic effects of different chemotherapeutic agents. Alterations in the expression of microRNAs are regulated by different factors, such as transcription factors (p53, PPARγ, and SAMD4) and epigenetic factors (hypermethylation and acetylation). Up to 158 microRNAs showed altered expression in pancreatic adenocarcinoma, such as overexpression or underexpression, which act together to lead to tumour growth, invasion, chemotherapy resistance, or epithelial–mesenchymal transition [88,89,90]. Different microRNAs were implicated in the suppression of oncosuppressive genes. For example, miR-21 acts on PTEN to downregulate or alter the expression of cyclin-dependent kinases, which lead to a loss of cell cycle control favouring tumour growth [91]. The overexpression of miR-424-5p led to a decrease in the expression of SOCS6 and stimulation of the Ras-ERK pathway, which stimulate cell growth [92]. An increase in miR-155 inhibits crucial genes in cell control, such as TP53 [93]. Underexpression of miR-203 leads to a loss of regulation of cell proliferation in the G1 phase due to alterations in SOCS3, which favours neoplastic proliferation [94]. Other molecules, such as miR-143, let-7-d, and miR-126, stimulate the expression of oncogenes, such as KRAS, and favour growth, cell migration, and metastatic invasion [95,96,97]. The epithelial–mesenchymal transition of pancreatic adenocarcinoma favours tumour growth at the tissue level, reinforces local immunosuppression, stimulates mechanisms of chemo- and radioresistance, and favours metastatic dissemination [98]. The set of microRNAs of the miR-200 family, such as miR-141, miR-429, miR-203, or miR-34b, is associated with the stimulation of epithelial–mesenchymal transition, angiogenesis, and lymphangiogenesis, which promote the presence of metastatic niches where tumour cells are later deposited [99]. The mechanism by which different microRNAs stimulate tissue invasion and metastasis are widely described as miRNA 218-, miR-155-, and miRNA-10-stimulating epidermal growth factors, TGF-β, metalloproteinases, and endothelial growth factors and the inhibition of metastasis suppressor genes, such as EP300, and they act together to promote the vascular invasion of tumour cells [100,101]. Chemoresistance in pancreatic adenocarcinoma is another limitation of the chemotherapeutic approach, and different microRNAs, such as miRNA-146 and miRNA 205, have been described, which stimulate the expression of oncogenes that provide chemoresistance to gemcitabine. Therefore, the roles of microRNAs in metastasis, tumour growth, and chemoresistance are broad [102,103]. Markers, such as miR-21, have prognostic value because their presence indicates a worse response to gemcitabine and worse prognosis [104].

MicroRNAs are also useful diagnostic markers. For example, Bloomston et al. used 25 microRNAs in 107 patients with pancreatic adenocarcinoma and chronic pancreatitis and obtained a sensitivity of 90% in the differentiation of pancreatic cells from chronic pancreatitis vs. pancreatic adenocarcinoma in numerous histological samples [105]. These markers may be used as serological markers, such as miR-221 or miR-1290, in the early diagnosis of this neoplasia and are measured in serological samples, which makes the diagnosis in earlier stages easier than diagnosis using CA19-9 [106]. Recent authors described the possible use of miRNAs as therapeutic targets in vivo and in vitro using viral release systems (e.g., miR145, miR143, and let-7 miR), which inhibit proliferation-inducing pathways, such as the KRAS pathway. Promitogenic kinases stimulate radiosensitivity by stimulating miR23b, which is decreased in tumour cells of pancreatic adenocarcinoma and favours the response to chemotherapy and radiotherapy [107,108,109].

The usefulness of miRNAs ranges from early diagnosis to possible therapeutic targets, and the large number of miRNAs makes research complex and generates a wide range of possibilities for the approach to this neoplasia.

There is an intrinsic relationship between microRNAs and exosomes. Exosomes are a set of extracellular vesicles between 30 and 100 nm in diameter that mediate the intercellular transport of microRNAs, messenger RNAs, coding DNAs, and another set of proteins that act as regulators and intercellular mediators of the tissue microenvironment and at a distance [110]. Within pancreatic cancer, these vesicles play a fundamental role in lymphangioproliferation, metastatic dissemination, and especially epithelial–mesenchymal transition. These exosomes participate in the transport of microRNAs, and the relationship between epithelial–mesenchymal transition and microRNAs was demonstrated [111,112]. For example, the downregulation of miR-429 and the overexpression of miR-361-3p favour the epithelial–mesenchymal transition from a locoregional point of view and stimulate the desmoplastic reaction, which generates local immunosuppression and stimulates tumour growth. [113,114] The clinical utility of exosomes and the microRNAs found within them is their detectability in different fluids, which favours early diagnosis and the ease of measurability. For example, Liu et al. demonstrated the usefulness of miRNA-16a and microRNA 196a with CA19-9 in the plasma of 140 patients with pancreatic adenocarcinoma vs. 179 control subjects or patients with chronic pancreatitis to differentiate patients with pancreatic neoplasms from patients without adenocarcinoma of the pancreas. They obtained diagnostic AUCs of 0.979 [115]. Other authors, such as Machida et al., compared exosomes containing miR-1246 and miR-4644 in patients with pancreatic adenocarcinoma to healthy controls and obtained AUCs of 0.814 for the differentiation of sick patients from healthy patients [116]. Most studies evaluated the diagnostic utility of exosomes in patients with adenocarcinoma compared to healthy controls.

Biopsy remains essential to the diagnosis of this disease in patients in advanced stages. The measurement of these serological markers in saliva and portal blood allows the screening of patients with risk factors, such as obesity, a strong family history of pancreatic adenocarcinoma, or intrapapillary mucinous tumours, or the detection of metastatic progression as a replacement of the currently used marker, CA19-9, which is elevated as a function of the tumour burden. However, it is not elevated in many cases until there is an advanced neoplasm, and it is never positive in the 5–10% of the population who do not have the Lewis antigen [117]. One of the main disadvantages of exosomes and microRNAs is the difficulty of standardising their nonpathological serological levels in different control populations compared to patients with adenocarcinoma and the fact that microRNAs are upregulated and downregulated in a multitude of diseases, which may interfere with other pathologies in their detection [118]. Therefore, the cost-effectiveness of these markers was evaluated recently, and their use in daily clinical practice is being analysed. Notably, microRNAs and exosomes are among the main molecules that may be used in the screening of pancreatic adenocarcinoma and provide information on the underlying physiopathology of a malignant neoplasm that is associated with a wide variety of locoregional histological alterations and a metastatic disease that threatens the current oncological management of these patients.

## 8. DNA Methylation

To address epigenetic alterations in the pathophysiology of pancreatic adenocarcinoma, we must remember the underlying histological alterations that characterise the progression from normal functional tissue to a true invasive neoplasia. First, numerous exogenous agents, such as tobacco, alcohol, and other substances, cause epigenetic alterations, hypomethylation of oncogenes, and hypermethylation of oncosuppressive genes, which alter the expression of different genes (p16, p53, DPC4, and BRCA2) and cause mutations in the KRAS gene during tissue change [119]. The KRAS mutation generates a series of modifications in the mechanisms of cellular repair and the regulation of growth due to its interaction with different growth factors (primarily TGF-β1 and FGF2) and signalling pathways, such as mitogen-activated protein kinase (MAPK) and phosphoinositide 3-kinase (PI3K)–Akt, which alter the cell proliferation of the tumour cell line, and other mutations (such as in the p16, p53, DPC4, and BRCA2 genes) and alterations in differentiation that eventually transform into a neoplastic lesion. [120,121] DNA methylation is catalysed by DNA methyltransferases, which add methyl groups to cytosine–guanine dinucleotides that involve the inactivation of oncosuppressive genes in addition to acting in the stimulation of mucins, such as MUC4 and MUC5AC, which correlate with an increased tendency towards invasion and tumour growth [122]. He et al. showed that alterations in the transcription factor GLI1 lead to alterations in the methyltransferases DNMT1 and DNMT3a, which favour the methylation of genes, such as p16, which is altered in 95% of patients with pancreatic adenocarcinoma, or KRAS [123]. The oncosuppressor p16 stimulates cyclin-dependent kinases that cause G1 phase progression and favour tumour growth [124]. Sato et al. found that claudin-4, lipocalin-2, 14-3-3σ, and trefoil factor 2 were overexpressed in histological samples of patients with pancreatic adenocarcinoma compared to healthy controls. [125] Alterations in the ultrastructure of histones in chromatin remodellers, such as p300, HDACs, and PBRM1, are altered in patients with pancreatic adenocarcinoma, which lead to the aberrant expression of c-MYC and KRAS, which act as oncogenes stimulating the proliferation of tumour cells in pancreatic adenocarcinoma. [126,127,128]

The complexity of epigenetic alterations in pancreatic adenocarcinoma allows us to understand the underlying pathophysiology of progression from a normal functional tissue to a properly invasive lesion. In the cost–benefit balance of tests to detect epigenetic alterations in daily clinical practice, it is much more interesting to focus on genetic alterations or other tissue or serological markers than on epigenetic alterations.

## 9. Instability of Microsatellites

Within tumours of the digestive system, the microinstability of satellites, which is an alteration in the repair system due to DNA mismatch, leads to the accumulation of a great variety of mutations (primarily in the MLH1, PMS2, MSH2, and MSH6 genes) that are detectable using immunohistochemistry, and DNA polymerase generates alterations in the length of the microsatellites (i.e., in consecutive repeats of noncoding DNA) in tumour cells. [129,130] These alterations were observed in hereditary neoplasms, such as hereditary nonpolyposis colon cancer and Lynch syndrome and sporadic colon cancers, where their diagnostic and therapeutic implications are a key advance in colon oncology. Microsatellite instability was found in up to 45% of patients with sporadic colon cancer, depending on the population and study [131]. The usefulness of this genetic marker is that it is associated with a better prognosis, longer disease-free survival, and a lower tendency to generate metastasis in colorectal cancer [132]. Different authors showed a prevalence of microsatellite instability of approximately 1–3% in pancreatic adenocarcinoma, which was associated with genes, such as KRAS, wild-type TP53, JAK, or KMT2 [133,134]. Different authors studied its usefulness because the FDA approved pembrolizumab for use in patients with neoplasms with alterations in microsatellites, but its usefulness in patients with pancreatic adenocarcinoma was limited in the clinical trial KEYNOTE-158 because only 4/22 patients with pancreatic adenocarcinoma showed a response [135]. Therefore, microsatellite instability has limited clinical and diagnostic utility in pancreatic adenocarcinoma, which demonstrates the complexity of the response to another possible immunotherapeutic agent in these cancer patients.

Two clinical trials (NCT00556023 or NCT02331251) are currently evaluating immunotherapy with anti-CTLA4 or anti-PDL1 but demonstrated a limited therapeutic effect in these patients. Although alterations in microsatellite instability have been studied in different digestive neoplasms and demonstrated utility in establishing prognostic factors, the same alterations do not occur in pancreatic adenocarcinoma due to its peculiarities (e.g., multiple accumulated mutations, epithelial–mesenchymal transition, and chemoresistance), which makes alterations in microsatellite instability within digestive tumours respond uniquely.

## 10. Genetic Alterations

The utility of genetic alterations elucidates the pathophysiology of pancreatic adenocarcinoma and its therapeutic implications. Most patients with pancreatic cancer have sporadic cases, without inheritable genetic alterations, but up to 10% of all pancreatic adenocarcinomas have a family history of this neoplasm [136]. Numerous mutations were described, but the most relevant involve alterations in the cell cycle, inhibition of apoptosis, progression of cell growth, and changes in different metabolic pathways that cause tumour chemoresistance. The recent identification of genetic markers allows for intensive follow-up of patients with strong family histories or genetic diseases where adenocarcinoma plays a leading role in the expression of the disease phenotypes. The correct heterogeneous identification of markers has allowed the study of new chemoimmunotherapy regimens in candidate patients and the identification of patients at higher risk of progression.

### 10.1. BRCA1/BRCA2

The BRCA1 gene is an oncosuppressor gene located on chromosome 17 that regulates the cell cycle by tightly regulating the repair of homologous DNA that has been altered. Therefore, deficiencies in homologous repair lead to genetic instability and alterations in different phases of the cell cycle. Mutations of the BRCA1 and BRCA2 genes are associated primarily with breast and ovarian cancer. Patients with BRCA-positive ovarian and metastatic breast cancer have a greater response to platinum-based chemotherapy and poly[ADP-ribose] polymerase inhibitors, such as olaparib [137]. Different studies tried to establish the prevalence of BRCA mutations in patients with pancreatic adenocarcinoma and found that up to 5.7% of patients had BRCA2 mutations and up to 2.3% had BRCA1 mutations. Up to 4.6% of patients had mutations in both genes [138,139]. The prevalence of BRCA mutations in patients of Ashkenazi origin was up to 19% (approximately 1/40 people had mutations in a BRCA gene in this population group, which explains their high prevalence of BRCA in pancreatic cancer) [140]. The National Comprehensive Cancer Network guidelines for the screening of patients with different mutations associated with an increased risk of pancreatic adenocarcinoma are candidates for screening programmes using contrast magnetic resonance or endoscopic ultrasound [141]. The therapeutic implications in patients with mutations of the BRCA genes have been studied. Golan et al. examined 71 patients treated for metastatic pancreatic adenocarcinoma, and a mean survival of 22 months was observed in patients with mutated BRCA genes who were treated with platinum compared to a mean survival of 9 months in patients treated with platinum-free chemotherapy [142]. Pokataev performed a meta-analysis of several studies and concluded that platinum-based chemotherapies were effective in patients with metastatic adenocarcinoma and mutations in a BRCA gene, with a tendency to exhibit a higher average survival in this subgroup of patients. However, clinical trials should be performed that correctly type these patients (e.g., germinal or somatic mutations, number of BRCA mutations, and mutations in genes related to deficiency in homologous recombination) to reach firmer conclusions about standard chemotherapeutic treatment vs. platinum-based therapies [143]. The use of poly(ADP polymerase) (PARP) inhibitors in breast or metastatic ovarian neoplasms with BRCA mutations yields better average survival [144]. Therefore, numerous authors studied the use of PARP inhibitors in patients with metastatic adenocarcinoma and BRCA gene mutations and observed higher average survival rates under maintenance therapy for metastatic adenocarcinoma. These results led to FDA approval of the use of olaparib as maintenance therapy in metastatic adenocarcinoma with an altered BRCA gene that responded to platinum-based chemotherapy [145]. Numerous clinical trials are evaluating other PARP inhibitors in these patients, such as NCT02184195 and NCT04548752.

Despite being present in a small percentage of patients, BRCA mutations in pancreatic adenocarcinoma have prognostic and therapeutic implications and allow us to understand and design therapeutic targets that will improve the average survival of these patients.

### 10.2. CDKN2A/p16

The p16 gene, also known as CDKN2A, is an oncosuppressive gene located on chromosome 9 that inhibits phosphorylation by cyclin-dependent kinases of different growth and proliferation factors, such as Rb and E2F, in the G1 phase. Therefore, alterations in this gene lead to hyperphosphorylation of the Rb protein, which affects the G1 phase and promotes cell growth and tumorigenesis. [146]. Notably, mutations in the p16 gene were found in up to 50% of patients with adenocarcinoma of the pancreas. [147] In patients with adenocarcinoma of the colon, non-small-cell lung cancer, or epidermoid of the larynx, mutations of the p16 gene are associated with a worse prognosis and higher recurrence rate. [148,149,150] At the immunohistochemical level, the correlation between the expression of p16 and mean survival is not conclusive. Some studies indicated that the negative expression of p16 was associated with worse survival, and other studies found no relationship between the expression of p16 and worse average survival. Gu et al. performed a meta-analysis and found that the overexpression of p16 did not reach statistical usefulness in predicting prognosis. [151] Because the relationship of p16 with pancreatic adenocarcinoma has been studied for many years, implications as a prognostic marker have been found. However, these data are of limited use because numerous studies did not demonstrate a correlation between its immunohistochemical level and prognosis. This marker allows us to understand one of the fundamental alterations in the aetiopathogenesis of pancreatic adenocarcinoma.

### 10.3. KRAS

The most frequent genetic alteration in pancreatic adenocarcinoma, which is present in 70–95% of patients, is KRAS mutation. KRAS is an oncogene whose activation causes a series of changes in metabolic pathways and stimulation of different intracellular growth factors and transcription factors, which lead to the stimulation of tumour cell proliferation that favours invasion, migration, and metastatic dissemination [152]. All of these processes start in the early, preinvasive stages when different mutations are acquired, and the final outcome is invasive adenocarcinoma. Up to 80 different altered metabolic pathways result from KRAS mutation, such as activation of MAPK and PI3K-AKT, which constitute one of the main altered metabolic pathways, and the expression of transcription factors, such as ELK, JUN, and MYC [153,154]. Approximately 95% of pancreatic adenocarcinoma patients are positive for KRAS mutation on endoscopy-guided or transcutaneous biopsy [155]. Different authors studied the clinical utility of PCR sequencing of liquid biopsies, such as peripancreatic fluid or circulating tumour cells. For example, Dabritz et al. analysed the presence of KRAS in the plasma of 56 patients with pancreatic adenocarcinoma and the expression of CA19-9 and obtained a sensitivity of 91% [156]. Cohen et al. analysed 221 patients with resectable pancreatic adenocarcinoma, and the serum KRAS level in combination with other biomarkers, such as osteopontin, CA19-9, and circulating DNA, obtained a sensitivity of 64% and a specificity of 99.5% [157]. Therefore, we can measure KRAS to identify pancreatic lesions after endoscopy-guided fine-needle biopsy and in peripheral blood in early disease stages. Other authors showed that the presence of KRAS in the tumour tissue itself or in plasma was associated with a worse prognosis with a higher rate of recurrence and worse average survival. [158,159]

The therapeutic usefulness of this marker is its utility as a therapeutic target. The mechanism by which KRAS protein expression may be blocked is via small interfering RNA. Small interfering RNAs delivered to pancreatic adenocarcinoma decreased tumour size and proliferation in vivo [160]. Golan et al. studied RNA interference using LOcal Drug EluteR (LODER), a biodegradable polymer that stabilizes RNA inference, in combination with FOLFIRINOX in a phase 1/2a clinical trial of 15 patients and observed a decrease in tumour size, improvement in CA19-9 level, and a decrease in tumour progression with a mean survival of 15.12 months [161].

The KRAS gene has high value in the diagnosis, prognosis, and possible future therapeutic regimens in patients with pancreatic adenocarcinoma.

### 10.4. p53

The TP53 gene is an oncosuppressive transcription factor that participates in apoptosis by activating several genes, such as BAX and PUMA, controlling the G1/S phase of the cell cycle by cyclin-dependent kinases, such as CDKN1A/p21, and activating DNA repair mechanisms, such as endonucleases, DNA polymerase, and other repair proteins, such as GADD45. [162,163] A mutation in the TP53 gene causes alterations in proliferation and promotes tumour growth. Up to 70% of pancreatic adenocarcinoma patients have TP53 mutations [164]. Altered p53 activity promotes the epithelial–mesenchymal transition due to the activation of cancer-associated fibroblasts, which generates locoregional dysplastic changes and causes locoregional immunosuppression [165]. Weismueller et al. studied the relationship between alterations of the TP53 gene and PDGFRβ (platelet-derived growth receptor beta) in the metastatic capacity of pancreatic adenocarcinoma cells in vitro, which were also related to a worse prognosis in vivo [166]. The prognostic implications of this marker were studied previously. For example, Xiang et al. showed that patients with alterations in the TP53 gene, whether due to mutations causing underexpression of the normal gene or a nonfunctioning TP53 gene, had an increase in tumour recurrence after pancreatectomy [167]. However, alterations of the TP53 gene induced chemiradial resistance by altering the expression of multidrug resistance gene (MDR) 1 and NRF2, which induce the elimination of chemotherapies, such as cisplatin [168].

The different studies on the p53 gene as a possible therapeutic target implicate complex molecular pharmacological mechanisms. APR-246 was studied as a methylated analogue of PRIMA that restored the functions of p53, and it is being studied in combination with other chemotherapeutic regimens in patients with ovarian cancer or oesophageal cancer in different clinical trials (NCT02098343, NCT02999893) [169]. Other molecules, such as ganetespib and histone deacetylase inhibitors, were also studied in non-small-cell lung neoplasms and gynaecological tumours and did not provide applicable benefits in daily clinical practice. [170,171]

Markers carry diagnostic implications and allow better understanding of the physiological alterations that lead to increased tumour spread and recurrence after surgical resection with curative intent. However, the possible therapeutic implications of p53 are complicated, and the difficulty is in the design of pharmacological therapies that act adequately in these patients.

### 10.5. DPC4/SMAD4

DPC4 is an oncosuppressor that is altered by different molecular pathways that involve TGF-β, and it is present in up to 80% of pancreatic adenocarcinomas. DPC4 and SMAD4 interact with SMAD regulatory receptors, which are transcription factors that regulate cell growth, cell fibrosis, and epithelial–mesenchymal transition. Therefore, alterations in these genes promote tumour cell growth, invasion, and desmoplastic reactions and epithelial–mesenchymal transition [172]. Shin et al. analysed the inactivation of this gene in 641 patients with pancreatic adenocarcinoma using immunohistochemistry and found that it was associated with a higher rate of locoregional recurrence after tumour resection, a higher probability of metastatic disease, and worse average survival [173]. Donahue et al. showed that the alteration of these genes in patients with metastatic adenocarcinomas was related to more aggressive neoplasms and worse average survival rates [174]. These results were confirmed in the meta-analysis of Du et al., who analysed 4247 patients in different studies of different neoplasms, including pancreatic adenocarcinoma. They concluded that loss of immunohistochemical expression of SMAD4 correlated with worse survival [175].

Due to the causal relationship between alterations of SMAD4 and TGF-β, the possible therapeutic utility of the latter was analysed. Using AP-120009, an antisense oligonucleotide that affects the messenger RNA of TGF-β2, Schlingensiepen et al. observed a decrease in tumour cell growth in vitro in human pancreatic tumour cells [176]. Oettle et al. observed partial responses to AP-120009 in a clinical trial, but they did not reach relevant conclusions, which suggests that larger studies are needed on the use of AP 12009 in patients with pancreatic adenocarcinoma or melanoma who exhibit acceptable pharmacological responses [177].

DPC4 is a prevalent genetic marker with relevant prognostic implications. As with the other genetic markers, the possibility of its use as a therapeutic target in pancreatic adenocarcinoma presents serious difficulties that prevent it from application in daily clinical practice.

## 11. Concluding Remarks

**Figure 1:**
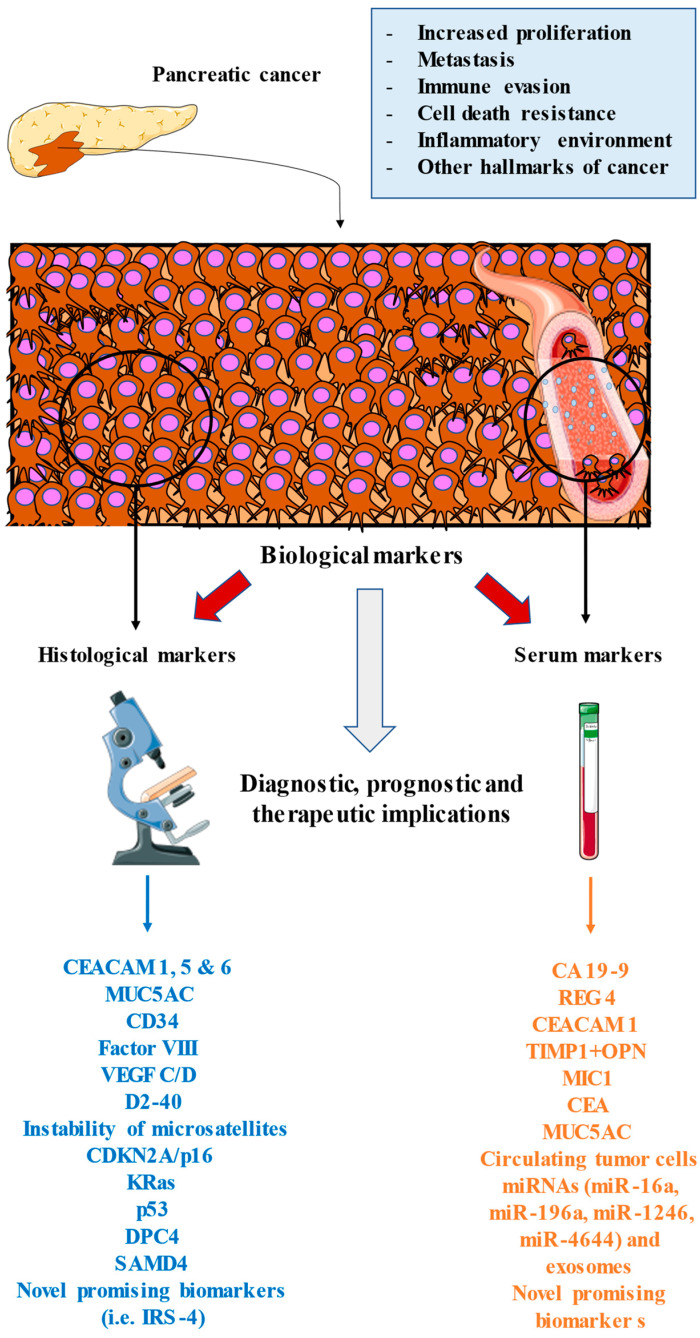
An overview of the main biomarkers used in pancreatic cancer. These markers are found in the proper tumour environment, as studied by histological techniques or released in blood or lymphatic vessels, which may be studied in the form of serological analysis. All of these markers are involved or related to the different oncogenic processes that occur in the tumour, including enhanced proliferation, immune and cell death evasion, the inflammatory environment, metastasis, and other hallmarks of cancer. The study and inclusion of these markers in clinical practice may greatly aid the clinical management of patients with pancreatic cancer, including at the diagnostic, prognostic, and therapeutic (predictive) levels.

Pancreatic cancer is a growing and potentially lethal cancer entailing multiple clinical difficulties. The usefulness of searching for novel histological and serological markers in pancreatic adenocarcinoma has led to a revolution in the diagnosis of these patients [178,179]. CA19-9 has been the marker of choice in daily clinical practice, but numerous new markers have allowed more correct staging, better prediction of prognosis, and more accurate diagnosis of pancreatic adenocarcinoma, which is an aggressive disease that is hard to detect in the early stages. As summarised in Figure 1, the introduction of these serological and histopathological markers provides multiple benefits to the clinical management of patients with pancreatic cancer, especially at the diagnostic or prognostic levels. Some of these markers have allowed the design of targeted therapies that are being tested, but we hope to promote the introduction of immunotherapy or regulators of metabolic pathways that can change the treatment paradigm of these patients. The most important biomarkers summarised in this manuscript are listed in Table 1 with their diagnostic, prognostic, and therapeutic/predictive utility.

## Figures and Tables

**Table 1:** A summary of the main biomarkers in pancreatic adenocarcinoma.

Marker	Type of Marker	Diagnostic Utility	Prognostic Value	Therapeutic/Predictive Value	References
CA 19-9	Serological	Yes, AUC = 0.878	Serological levels of CA19-9 are related to higher tumour burden	No	[10]
REG 4	Serological	Yes, AUC = 0.922	No	No	[19]
CEACAM	Histopathological/Serological	Yes AUC = 0.948 in combination with CA19-9	Higher expression levels correlate with a poorer prognosis	No	[20,21]
TIMP1 + OPN	Serological	Yes, sensitivity power at 89.5%	Higher expression levels correlate with a poorer prognosis	No	[22]
MIC1	Serological	Yes, AUC = 0.886	Higher expression levels correlate with increased recurrences after curative surgery	No	[27]
CEA	Serological	Yes, AUC = 0.900	Higher expression levels correlate with a poorer prognosis	No	[31]
MUC5AC	Serological/Histopathological	Yes, AUC = 0.894 in combination with CA 19-9	Higher expression levels correlate with a poorer prognosis	No	[43]
CD34/FVIII	Histopathological angiogenesis	Yes	Higher expression levels correlate with a poorer prognosis	Possible application of antiangiogenic drugs that is being evaluated	[62,63,64,65,66,67]
VEGF C/D D240	Histopathological	Yes	Higher expression levels correlate with a poorer prognosis	No	[68,69,70]
Circulating tumour cells (CTCs)	Serological	Sensitivity: 84%	Higher tumour cell detection correlates with a poorer prognosis	No	[81,82,83]
miRNA-16a + miRNA196a	Serological	AUC = 0.979 in combination with CA 19-9	No	No	[115]
miRNA 1246 + miRNA4644	Serological	AUC = 0.814	No	No	[116]
Microsatellite instability	Histopathological	Limited; 1–3% of patients present alterations	Inconclusive	Limited therapeutic implications	[133,134]
BRCA1/BRCA2	Genetic Histopathological	BRCA1 = 2.3% patients. BRCA2 = 5.7% patients. BRCA1 + BRCA2 = 4.6% patients	Poorer prognosis	Possible greater response to platinum-based chemotherapies; response to PARP inhibitors in metastatic adenocarcinomas	[138,139,140,141,142,143,144,145]
CDKN2A/p16	Genetic Histopathological	50% of patients with pancreatic adenocarcinoma show this alteration	Current studies are contradictory	No	[147,151]
KRAS	Genetic Histopathological Serological	95% of patients with pancreatic adenocarcinoma show this alteration Sensitivity: 64% Specificity: 99.5% in combination with Ca19-9	Higher tumour cell detection correlates with a poorer prognosis	Possible use of RNA interference by Local Drug Eluter	[156,157,158,159,160,161]
p53	Genetic Histopathological	70% of patients with pancreatic adenocarcinoma	Associated with tumour’s aggressiveness	Possible use of APR-246 or ganetespib to restore P53 functions with limited therapeutic effects. Linked with cisplatin resistance	[164,167,168,169]
DPC4/SAMD4	Genetic Histopathological	80% of patients with pancreatic adenocarcinoma	Higher rate of locoregional recurrence after tumour resection, greater probability of metastatic disease and reduced survival	Possible use of AP-120009 with limited therapeutic effects	[172,173,174,175,176,177]

## Data Availability

Not applicable.
